# Supplementary material for: Reconfigurable and responsive droplet-based compound micro-lenses
Source: Nat Commun. 2017 Mar 7;8:14673. doi: 10.1038/ncomms14673 (PMC5344304; doi:10.1038/ncomms14673)
Supplement: Supplementary Information — Supplementary Figures, Supplementary Notes and Supplementary References [file ncomms14673-s1.pdf]

Supplementary Information

Supplementary Figures

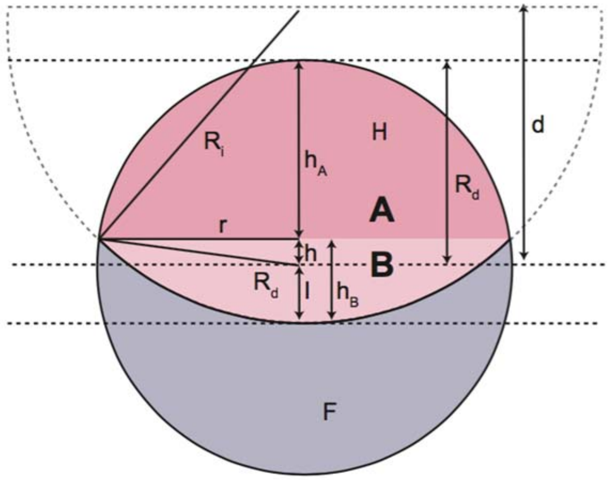

Supplementary Figure 1: Geometry of Double Emulsion Droplet.

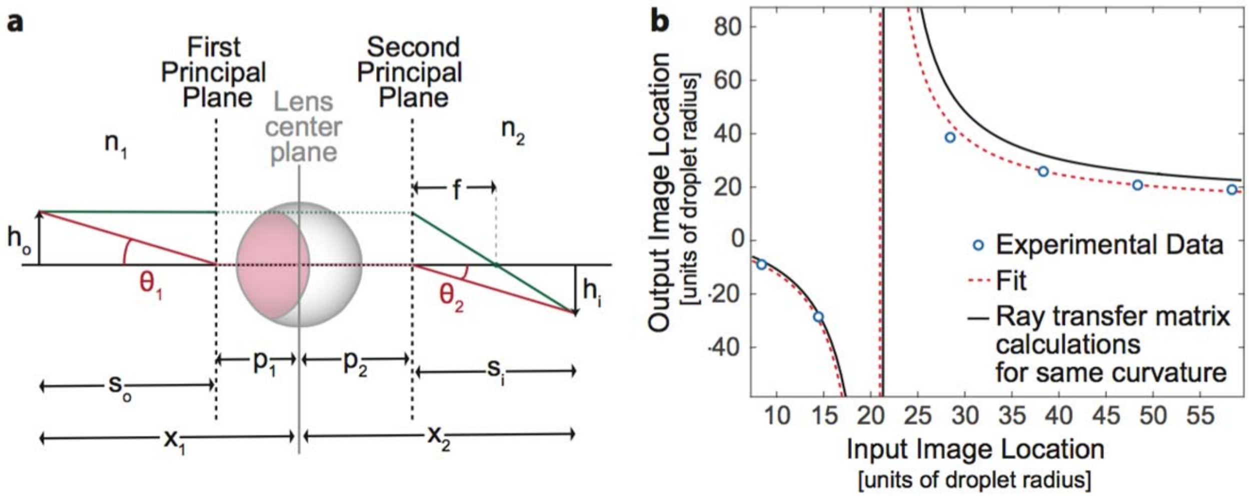

Supplementary Figure 2: Determination of the focal length from the image location. a) Ray Diagram for thick lens located at the edge of two different media. **b)** Example plot of the image location used to determine focal length. The dashed red line is the least squares fit to Eq. 5 in the main text with fitting parameters $f = 13.8 \pm 0.4$, $p_1 = 2.2 \pm 0.5$, and $p_2 = -2.2 \pm 0.9$ in units of droplet radius. The solid black line shows the expected image locations using the ray transfer matrix for a droplet with internal radius of curvature $R_{\mathrm{i}} = 2.03$ (determined from the side view of the droplet) and volume ratio v_{r}=1.

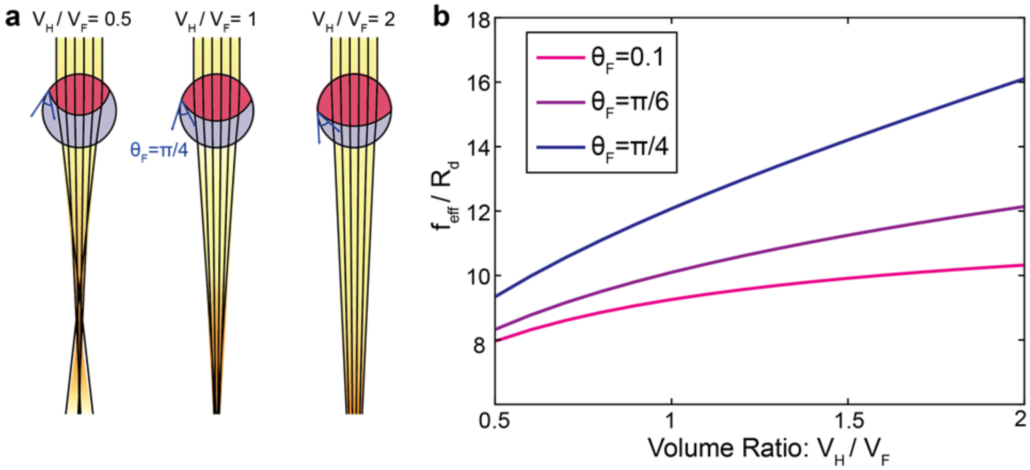

Supplementary Figure 3: Variation of focal length as a function of volume ratio. **a)** Ray tracing diagrams for droplets with volume ratios of hydro- to fluorocarbon $V_{\mathrm{H}}/V_{\mathrm{F}} = 0.5$, 1, and 2. Here, the contact angle measured from the hydrocarobon-fluorocarbon interface to the fluorocarbon-water interface is kept constant at $\theta_{\mathrm{F}} = \pi/4$. **b)** Effective focal length in units of droplet radius $f_{\mathrm{eff}}/R_{\mathrm{d}}$ plotted against volume ratio $V_{\mathrm{H}}/V_{\mathrm{F}}$, for contact angles θ_{F} of 0.1, $\pi/6$, and $\pi/4$.

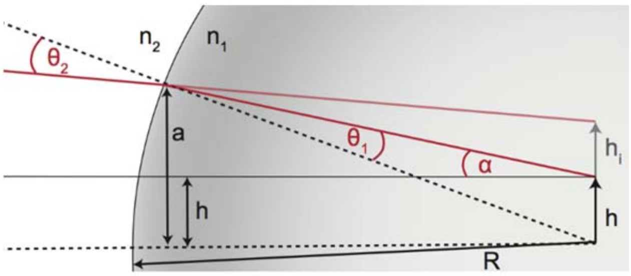

Supplementary Figure 4: Location of the image inside of a droplet using the Paraxial Approximation.

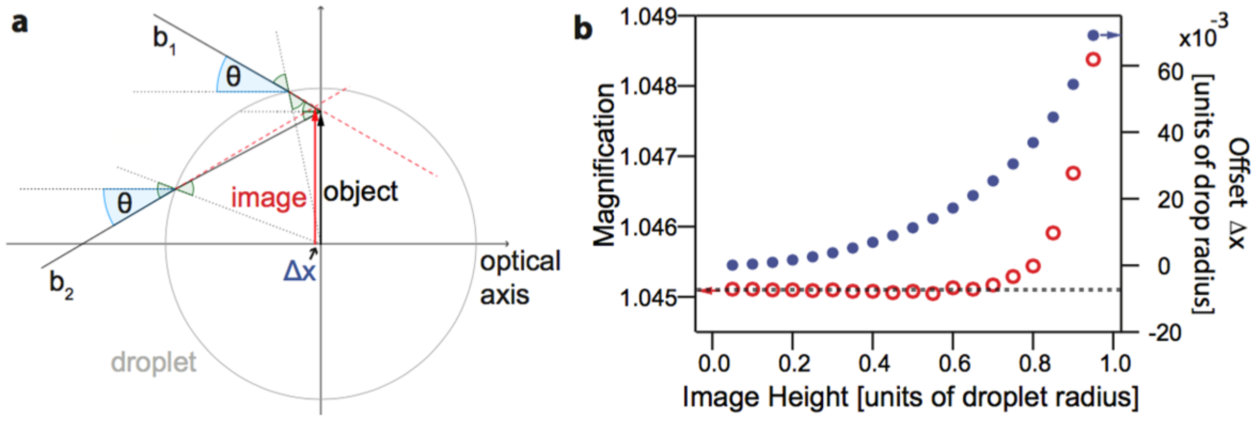

Supplementary Figure 5 Magnification within droplets without using ray tracing. a) Geogebra[52] model used to determine the apparent height of an object located inside a droplet of refractive index n = 1.39. Rays b_1 and b_2 signify light rays propagating at maximum collection angle θ given by the imaging objective's numerical aperture. **b)** Magnification of the object located inside of the droplet (red circles) and difference in position between object and image Δx (dark blue circles). The dashed black line shows the paraxial approximation magnification for the same droplet.

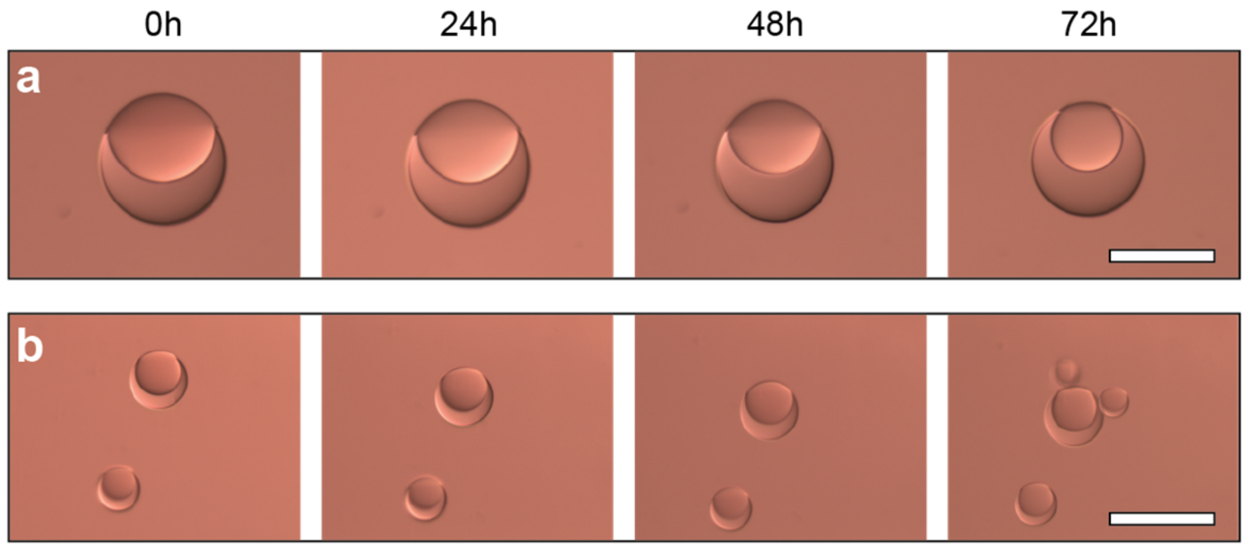

Supplementary Figure 6: Droplet stability. **a)** Variation of droplet morphology composed of heptane and FC770 over time, if diffusion of heptane is not suppressed. **b)** Droplet morphologies are stable, if diffusion of heptane is suppressed by enclosing the system and priming the aqueous medium with heptane. In the last frame shown in (b) two smaller droplets that also appear stable have drifted into the frame. Scale bars: 100µm.

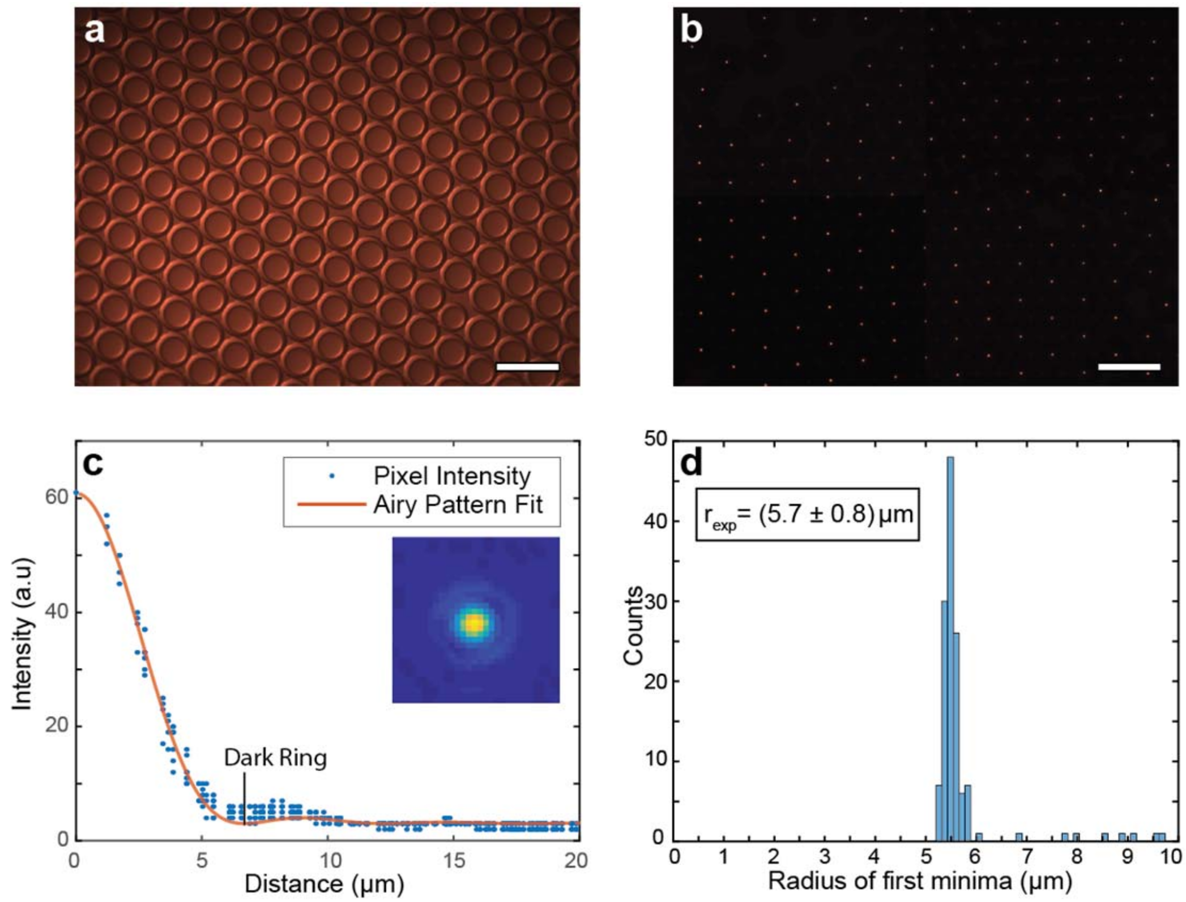

Supplementary Figure 7: Droplet homogeneity and point spread function uniformity. a) An array of uniform double emulsion droplet lenses. **b)** Point spread functions (PSF) of the droplets shown in (a). Scale bars: 500µm **c)** Fit to the Airy disk. d) Distribution of the minimum in the Airy pattern, which is a measure of the Rayleigh two-point resolution limit. The radius of the point spread function imaged through the 4x objective was (5.7 ± 0.8) µm. The larger size of the airy disk for these measurements is due to the fact that the point spread function of the droplets is convolved with that of the objective. A 4x objective was used to image a larger amount of droplets.

Supplementary Notes

Supplementary Note 1: Drop Geometry: Derivation of Equations (1) and (2) in the Main Text

The morphology of the droplet with heptane as the internal phase is determined by the surface tensions of the various liquids and the volume ratio $v_\mathrm{r} = \frac{V_\mathrm{H}}{V_\mathrm{F}}$, where V_H is the volume of heptane and V_F the volume of FC-770. If the overall droplet is considered to be spherical, then the volume of heptane is:

$$V_\mathrm{H} = \frac{v_\mathrm{r}}{1+v_\mathrm{r}} V_\mathrm{drop} = \frac{v_\mathrm{r}}{1+v_\mathrm{r}} \frac{4}{3} \pi R_\mathrm{d}^3 \, , \quad (1)$$

with R_d being the droplet radius (Fig. S1). The location of the interface was determined from the internal curvature R_i, by noting that the volume of heptane is equal to the volume of region A plus the volume of region B, each of which can easily be obtained by calculating the volumes of the spherical caps:

$$V_\mathrm{H} = V_\mathrm{A} + V_\mathrm{B} = \frac{\pi}{3} h_\mathrm{A}^2 (3R_\mathrm{d} - h_\mathrm{A}) + \frac{\pi}{3} h_\mathrm{B}^2 (3R_\mathrm{i} - h_\mathrm{B}) \quad (2)$$

The height of the spherical caps is determined by the distance from the center of the droplet to the plane of the three-phase contact line h, such that:

$$h_\mathrm{A} = R_\mathrm{d} - h \quad \text{and} \quad h_\mathrm{B} = l + h. \quad (3)$$

The location of the three-phase contact line is the intersection of the sphere forming the internal interface with the sphere that defines the overall droplet. In other words,

$$r^2 + h^2 = R_\mathrm{d}^2 \quad \text{and} \quad r^2 + (d - h)^2 = R_\mathrm{i}^2 \,, \quad (4)$$

where d is the distance from the center of the droplet to the center of the sphere that defines the internal interface.

Combining Supplementary Eqs. 1-3 and eliminating $r, h, h_\mathrm{A}, h_\mathrm{B}$, and d, yields Eq. 1 in the main text.

In the limit that $\gamma_\mathrm{FH} \ll \gamma_\mathrm{F} \approx \gamma_\mathrm{H}$, it has been shown that the contact angles at the three phase contact line are[1]

$$\frac{\gamma_\mathrm{H} - \gamma_\mathrm{F}}{\gamma_\mathrm{HF}} = \cos(\theta_\mathrm{F}) = -\cos(\theta_\mathrm{H}) \,, \quad (5)$$

which by applying the law of cosines yields Eq. 2 in the main text.

Supplementary Note 2: Determination of the Focal Length from the Image-Object Locations

In order to determine the droplets' focal length, a pattern was projected in front of the droplets and used as the "object". The image formed by the drops (the "image") was recorded. By varying the location of the object, the image location can be varied, allowing us to determine the focal length of the drops. A common form of the standard thick lens equation was used to find the focal length, accounting for the fact that the droplet-coverslip system formed a boundary between two different refractive index media, as shown in Fig. S2a.

Consider an object of height h_o located at a distance x_1 from the center of the droplet. For a thick lens system, all refraction can be considered to happen at the principal planes, such that a ray passing through the optical axis at the first principal plane (red ray in Fig S2a) will be refracted according to Snell's law, which in the paraxial approximation is given by:

$$n_1 \theta_1 = n_2 \theta_2 \,,$$

with n_1, n_2 and θ_1, θ_2 being the refractive indices and angles of the incident and refracted rays with the interface normal for medium 1 and 2, respectively. This can also be written in terms of the object and

image heights h_o, h_i, the distance of the object to the first principal plane s_o, and the distance of the image to the second principal plane s_i:

$$n_1 \frac{h_\mathrm{o}}{s_\mathrm{o}} = n_2 \frac{-h_\mathrm{i}}{s_\mathrm{i}} . \tag{6}$$

A ray that hits the first principal plane parallel to the optical axis will be refracted at the second principal plane and pass through the back focal point, which gives

$$\frac{h_\mathrm{o}}{f} = -\frac{h_\mathrm{i}}{s_\mathrm{i}-f} , \tag{7}$$

with f being the focal length measured in the second medium. Combining Supplementary Eqs. 6 and 7 yields a modified version of the lens equation:

$$\frac{n_1}{n_2}\frac{1}{s_\mathrm{o}} + \frac{1}{s_\mathrm{i}} = \frac{1}{f} .$$

In terms of the measured distances from the center of the droplet, this yields:

$$\frac{n_1}{n_2}\frac{1}{x_1-p_1} + \frac{1}{x_2-p_2} = \frac{1}{f} , \tag{8}$$

where p_1 and p_2 are the distances from the droplet center to the first and second principal planes, which were used as fitting parameters when determining the focal length of the droplets, as shown in Fig. S2b.

Supplementary Note 3: Correction to Interface Shape Due to Refraction at Droplet Interface

When the interface between the two liquids is imaged through the outer phase of the droplet, the image of the interface is magnified. In order to correct for the magnification, the paraxial approximation was used. Consider an object of height h located inside of a sphere of radius R and refractive index n_1, which is positioned inside a medium of refractive index n_2, as shown in Supplementary Figure 4. A ray leaving the object at an angle α will hit the interface of the drop at a height $a = h + R\alpha$ (under the assumption $\alpha \ll 1$,). At this height, the surface normal is at an angle $\frac{a}{R} = \frac{h}{R} + \alpha$, such that the ray hits the surface at an angle

$$\theta_1 = \frac{h}{R} + \alpha - \alpha = \frac{h}{R} \tag{9}$$

for all rays with $\alpha \ll 1$. Each ray is refracted according to Snell's law:

$$\theta_2 = \frac{n_1}{n_2}\theta_1 = \frac{n_1}{n_2}\frac{h}{R} \tag{10}$$

Similarly, the height were all the rays converge (the image is formed) is given by:

$$h_\mathrm{i} = R\theta_2 = \frac{n_1}{n_2} h . \tag{11}$$

The paraxial approximation breaks down for large angles, which is the case for a large object located inside a sphere. However, the magnitude of the error is small. Snell's law was used in the free software package Geogebra[2] to determine the image height of an object located inside a droplet of refractive index $n_1 = 1.39$ (heptane) in a medium of refractive index $n_2 = 1.33$ (water). Supplementary Figure 5 shows how the magnification depends on image height. For a small object, the magnification matches that of the paraxial approximation, but diverges for larger objects. For an object of 0.9 times the radius of the droplet, the error resulting from the paraxial approximation was less than 0.3%, which is significantly smaller than the uncertainty in the location of the interface (line thickness) in the images.

Supplementary Note 4: Vector Form of Snell's Law for Ray Tracing

The ray tracer that we implemented in MATLAB uses Snell's Law in vector form in order to unambiguously determine the direction of each ray after it was refracted through a surface. Consider a ray traveling along a vector $\vec{\mathbf{d}}_1$ refracting through a surface with normal $\vec{\mathbf{n}}$. The incidence angle θ_1 is given by the angle between the propagation vector $\vec{\mathbf{d}}_1$ and the surface normal $\vec{\mathbf{n}}$. The vector $\vec{\mathbf{d}}_1$ of the incident light can be broken down into components tangential ($\vec{\mathbf{t}}$) and normal ($\vec{\mathbf{n}}$) to the surface:

$$\vec{\mathbf{d}}_1 = \sin(\theta_1)\vec{\mathbf{t}} + \cos(\theta_1)\,\vec{\mathbf{n}}\,. \quad (12)$$

Similarly the propagation vector $\vec{d}_2$ of the outgoing ray can be written as

$$\vec{\mathbf{d}}_2 = \sin(\theta_2)\vec{\mathbf{t}} + \cos(\theta_2)\,\vec{\mathbf{n}}\,, \quad (13)$$

where θ_2 is the angle between $\vec{\mathbf{d}}_2$ and $\vec{\mathbf{n}}$.

The tangential vector $\vec{t}$ can be determined from Supplementary Eq. 12:

$$\vec{\mathbf{t}} = \frac{\vec{\mathbf{d}}_1 - \cos(\theta_1)\vec{\mathbf{n}}}{\sin(\theta_1)} \quad (14)$$

The outgoing angle θ_2 can be determined from Snell's law:

$$n_1 \sin(\theta_1) = n_2 \sin(\theta_2). \quad (15)$$

Using the trigonometric identity $\sin^2\theta + \cos^2\theta = 1$ Snell's Law can be written as

$$\cos^2(\theta_2) = \sqrt{1 - \left(\frac{n_1}{n_2}\right)^2 [1 - \cos^2(\theta_1)]} \quad (16)$$

Substituting Supplementary Eqs. 14-16 into Supplementary Eq. 13 yields the vector form of Snell's law

$$\overrightarrow{\mathbf{d}_2} = \frac{n_1}{n_2}\vec{\mathbf{d}}_1 + \left(\frac{n_1}{n_2}\cos(\theta_1) - \sqrt{1 - \left(\frac{n_1}{n_2}\right)^2 [1 - \cos^2(\theta_1)]}\right)\vec{\mathbf{n}}$$

Supplementary Note 5: Droplet stability

The solubility of hexane, heptane, and FC770 is extremely low in water; however, over sufficiently long time scales, diffusion of hexane or heptane through the aqueous medium into the ambient environment can lead to changes in droplet morphology. This is easily prevented by keeping the droplets and the medium in a closed environment and by suppressing diffusion into the aqueous medium, which can be achieved through priming with the respective solvent. FC770, a long-chain fluorinated oil, was not found to diffuse into the aqueous medium on the timescale of days. Supplementary Figure 6a visualizes droplet morphology variation in suboptimal experiment condition, while Supplementary Figure 6 shows that the droplets are stable, if the experimental conditions are appropriately controlled. For the heptane-FC770 droplets shown in the figure, we seal the sample chamber to prevent any exchanges with the ambient environment and saturate the aqueous medium with heptane, to avoid heptane diffusion from the droplets into the medium. Longer-term stability tests have not yet been conducted but will be addressed in future work. A rich parameter space regarding the chemical composition of the droplets remains to be explored, in order to optimize emulsion droplet compound micro-lenses for specific application scenarios. The droplets' chemical make-up affects their range of focal length, resolution capabilities, and will also influence such parameters as boiling points, pressure stability, compatibility with surrounding media and interfacial interactions.

Supplementary References

1. Guzowski, J., Korczyk, P. M., Jakiela, S. & Garstecki, P. The structure and stability of multiple micro-droplets. *Soft Matter* **8,** 7269–7278 (2012).

2. Hohenwarter, M. *et al. GeoGebra*. (IGI, 2007).

3. Zarzar, L. D. *et al.* Dynamically reconfigurable complex emulsions via tunable interfacial tensions. *Nature* **518,** 520–524 (2015).
